# Central odontogenic fibroma: retrospective study of six cases with variable histopathologic features using 2022 WHO classification

**DOI:** 10.1186/s12903-024-05085-w

**Published:** 2024-10-26

**Authors:** Sopee Poomsawat, Sirada Choakdeewanitthumrong, Jira Kitisubkanchana, Theerachai Kosanwat

**Affiliations:** 1 Department of Oral and Maxillofacial Pathology, Faculty of Dentistry, Mahidol University, Yothi Street, Bangkok, 10400 Thailand; 2 Department of Oral and Maxillofacial Radiology, Faculty of Dentistry, Mahidol University, Bangkok, Thailand

**Keywords:** Aneurysmal bone cyst, Central giant cell granuloma, Central odontogenic fibroma, Hybrid odontogenic lesions, Ossifying subtype

## Abstract

**Background:**

Central odontogenic fibroma (COF) is a rare benign odontogenic tumor with a wide range of histopathologic features. We evaluated COF diagnosed in our institute with 16 years’ experience using 2022 WHO classification.

**Methods:**

Our archives were reviewed and cases diagnosed as COF were selected. Clinical, radiographic and microscopic features were tabulated and analyzed.

**Results:**

Of 13,736 specimens, six cases (0.04%) of COF were discovered. Patients ranged in age from 14 to 44 years. There were two males and four females. Maxilla and mandible were affected equally. Five cases showed radiolucent appearance (4 unilocular, 1 multilocular) and one case showed a mixed radiolucent-radiopaque pattern. Histopathologically, three cases were conventional type (2 epithelium-rich, 1 epithelium-poor). Two cases were the hybrid COF with central giant cell granuloma (CGCG) and one of which was also associated with secondary aneurysmal bone cyst (ABC). The last case with a mixed radiolucent-radiopaque pattern was the ossifying subtype.

**Conclusion:**

Our results demonstrated that COF is a rare odontogenic tumor and exhibits diverse radiographic and microscopic appearances. The triphasic tumor consisting of the COF, CGCG and ABC, is reported here for the first time, while the ossifying subtype is considered the tenth case reported in the English-language literature. Oral and maxillofacial pathologists and other healthcare personnel must be aware of this rare odontogenic tumor and its variants to achieve the definite diagnosis.

**Clinical trial number:**

Not applicable.

## Background

Central odontogenic fibroma (COF) is a rare benign mesenchymal odontogenic tumor of the jawbone. COF accounted for 0.3 to 2.8% of odontogenic tumors based on studies containing a minimum of 1,000 odontogenic tumors [1–4]. According to the most recent systematic review, 135 cases of COF have been documented [5]. COF exhibits a slight female predilection and is frequently found in the second and third decades of life. The mandible and the maxilla are relatively equally affected. The molar region of the mandible and the anterior to the first molar area of the maxilla are the predilection sites. The most common clinical oral manifestation is asymptomatic swelling. Radiographically, COF typically manifests as a well-defined unilocular radiolucency (54%). Multilocular radiolucent appearance accounts for approximately 24% of COF. Approximately 11% of COF show a mixed radiolucent-radiopaque image [5].

Histopathologically, COF exhibits a wide range of features. The tumor is composed of variable amounts of inactive odontogenic epithelial rests distributed throughout the mature fibrous connective tissue. The stroma can range from myxoid to collagenized. COF also demonstrates varying degrees of cellularity from relatively acellular to moderately cellular. Calcifications with appearances of cementum-like material or dentinoid are present in some tumors [5–9]. COF was formerly classified in two subtypes. The first is the epithelium-rich type formerly known as the complex or the World Health Organization (WHO) type, characterized by prominent islands or strands of odontogenic epithelial rests. The second subtype is the epithelium-poor type previously known as the simple type, exhibiting minimal islands or cords of odontogenic epithelium. Additionally, tumors absence from odontogenic epithelium are also considered to be COF [10–12]. Although the histopathologic variations of COF have long been recognized, histopathologic variants were clearly described in the 2022 WHO classification [6, 13]. The subtypes included amyloid, granular cell, ossifying and hybrid COF with central giant cell granuloma (CGCG). In the previous WHO classification published in 2017, nearly all these subtypes were described as variants of COF [14]. These included the COF with amyloid-like protein deposition, the COF associated with CGCG and the granular cell odontogenic tumor. By contrast, the ossifying subtype has been introduced for the first time and is regarded as a new entity in the present WHO classification. Additionally, in the 2022 WHO classification, the amyloid subtype has been described in more details compared with the previous edition. In the present WHO classification, the amyloid subtype is characterized by amyloid deposits with Langerhans cells often found among the epithelial components. Notably, the amyloid subtype of COF remains a controversial tumor as it is still needed to distinguish from the non-calcifying Langerhans cell-rich of calcifying epithelial odontogenic tumor. Currently, there is no diagnostic criteria to separate these two entities. However, the site and gender predilection, biological behavior and immunohistochemical phenotypes of most controversial tumors are more similar to the amyloid subtype of COF [15, 16].

Due to the rarity of COF, most studies of COF were case reports or small series. At present, there are only 10 studies containing more than 10 cases of COF in their studies [9, 17]. They were published between 1990 and 2023. The largest series of COF comprises 62 cases, collecting data from 13 oral pathology laboratories located in 8 countries [9]. There were 43 females and 19 males with the average age of 33.9 years. Thirty-three cases were in the maxilla and 29 cases were found in the mandible. The unilocular radiolucency were more common than the multilocular pattern with a ratio of 2.25:1. The most common histopathologic feature was the epithelium-rich type (33 cases), followed by the amyloid variant (10 cases), the association with CGCG (7 cases) and the ossifying variant (6 cases). The other variants include the epithelium-poor type (3 cases) and the granular cell variant (3 cases). Taken all together, these findings indicate that COF is a rare odontogenic tumor and remains unfamiliar to pathologists. The diverse histopathologic features pose a significant challenge in diagnosing this tumor.

This study evaluated COF diagnosed in our institute with 16 years’ experience using 2022 WHO classification. The findings of this study may contribute to a better understanding of this rare odontogenic tumor and may assist oral and maxillofacial pathologists in developing a histopathologic diagnosis. Furthermore, the results can also benefit oral and maxillofacial radiologists, oral and maxillofacial surgeons and general practitioners in making clinical differential diagnoses.

## Methods

The archives of our institute between January 2008 and December 2023 were reviewed. Cases diagnosed as COF were selected. The criteria for diagnosis were based upon the latest WHO Classification of Head and Neck Tumours [6]. Cases that lacked histopathologic slides and radiographic images were excluded. Hematoxylin and eosin-stained sections of the chosen cases were reviewed by a Board-certified oral pathologist (SP). The final diagnosis was confirmed using two oral pathologists (SC, TK). Besides microscopic examination from the hematoxylin and eosin-stained slides, the diagnosis of one COF was confirmed by immunohistochemistry. Using the BOND Polymer Refine Detection System (Leica biosystems), slides were stained according to the manufacturers’ instructions with the following antibodies: CD99 (1:250, clone EP8; Cell Marque), S-100 (1:2000, clone 4C4.9; Cell Marque), SMA (1:600, clone 1A4; Cell Marque), Muscle specific actin (1:1000; clone HHF 35; Dako), Desmin (1:300, clone D33; Dako), CD31 (1:800, clone 7C70A; Dako), CD34 (1:800, clone QBEnd/10; Cell Marque), STAT-6 (1:50, clone EP325; Ward Medic), vimentin (1:500, cloneV9; Cell Marque) and cytokeratin cocktail (1:1200, clone AE1/AE3; Novocastra). In brief, new sections of four µm thickness were cut from paraffin-embedded blocks. Sections were deparaffinized and rehydrated before incubating in 3% H_2_O_2_ to block endogenous peroxidase activity. Antigen retrieval was performed by heating the sections in Ethylenediaminetetraacetic acid (EDTA) pH9.0 at 100^o^C for 25 min. Diaminobenzidine was used as the chromogen. Finally, sections were lightly stained with Mayer’s hematoxylin. Negative controls were obtained by leaving out the primary antibodies. Appropriate positive controls were used for individual antibody.

Clinical information including location, signs and symptoms, age and sex of the patients were recorded. Treatment modality as well as follow-up information were also collected. Radiographs of all cases were evaluated by a Board-certified oral radiologist (JK). The radiographic features regarding border, shape, radiodensity, associated impacted tooth, tooth/teeth displacement, root resorption, bone expansion and bone perforation were analyzed and tabulated. The border was defined as smooth or scallop. The shape was defined as unilocular or multilocular. The radiodensity was recorded as radiolucent, radiopaque or mixed radiolucent-radiopaque. The remaining radiographic features were recorded as yes or no.

This retrospective study followed the guidelines of the Human Research Committee of our institute, in compliance with the ethical standards of the Helsinki declaration (COA. No. MU-DT/PY-IRB 2023/011.3101).

## Results

During the 16-year period, only six cases of COF were discovered from 817 odontogenic tumors and 13,736 specimens. The clinical features of these six patients are displayed in Table 1. There were two males and four females. The average age was 27.8 years with an age range of 14 to 44 years. Maxilla and mandible were affected equally. In the maxilla, two cases occurred anterior to the first molar whereas one case extended from the canine to the tuberosity. In contrast to the maxilla, all lesions in the mandible occurred in the posterior region. The chief complaint of one half of these patients was swelling of the affected area. Case 1 did not present any symptoms whereas case 2 presented with toothache and a palatal depression. Case 6 visited the dental clinic to evaluate delayed tooth eruption of the permanent mandibular right second molar.

**Table 1:** Clinicopathologic features of 6 cases of central odontogenic fibroma

Case	Age (years)/ Sex	Location	Signs and symptoms	Microscopic pattern	Treatment	Outcome /Follow up (months)
1	35/F	L Max, C to 2nd P	Asymptomatic	Conventional, the epithelium-rich	Enucleation with TE (2nd P)	N/4
2	36/ M	R Max, 2nd I to 2nd P	Toothache, palatal depression	Conventional, the epithelium-rich	Enucleation with TE (1st P)	N/32
3	44/F	L Mand, 1st M to 3rd M	Swelling	Conventional, the epithelium-poor	Enucleation with TE (2nd M, 3rd M)	N/6
4	24/M	L Mand, 2nd P to 2nd M	Swelling	Hybrid COF with CGCG	Enucleation and curettage with TE (2nd P to 2nd M)	N/19
5	14/F	R Max, C to tuberosity	Swelling	Hybrid COF with CGCG with secondary ABC	Partial block resection, enucleation, and curettage	N/33
6	14/F	R Mand, 2nd M to ramus	Delay tooth eruption	Ossifying subtype	Enucleation and peripheral ostectomy with TE (2nd M)	N/22

M, Male; F, female; L, left; R, right; Max, maxilla; Mand, mandible; C, canine; I, incisor; P, premolar; M, molar; COF, central odontogenic fibroma; CGCG, central giant cell granuloma; ABC, aneurysmal bone cyst; TE, tooth extraction; N, no sign of recurrence

**Fig. 1:**
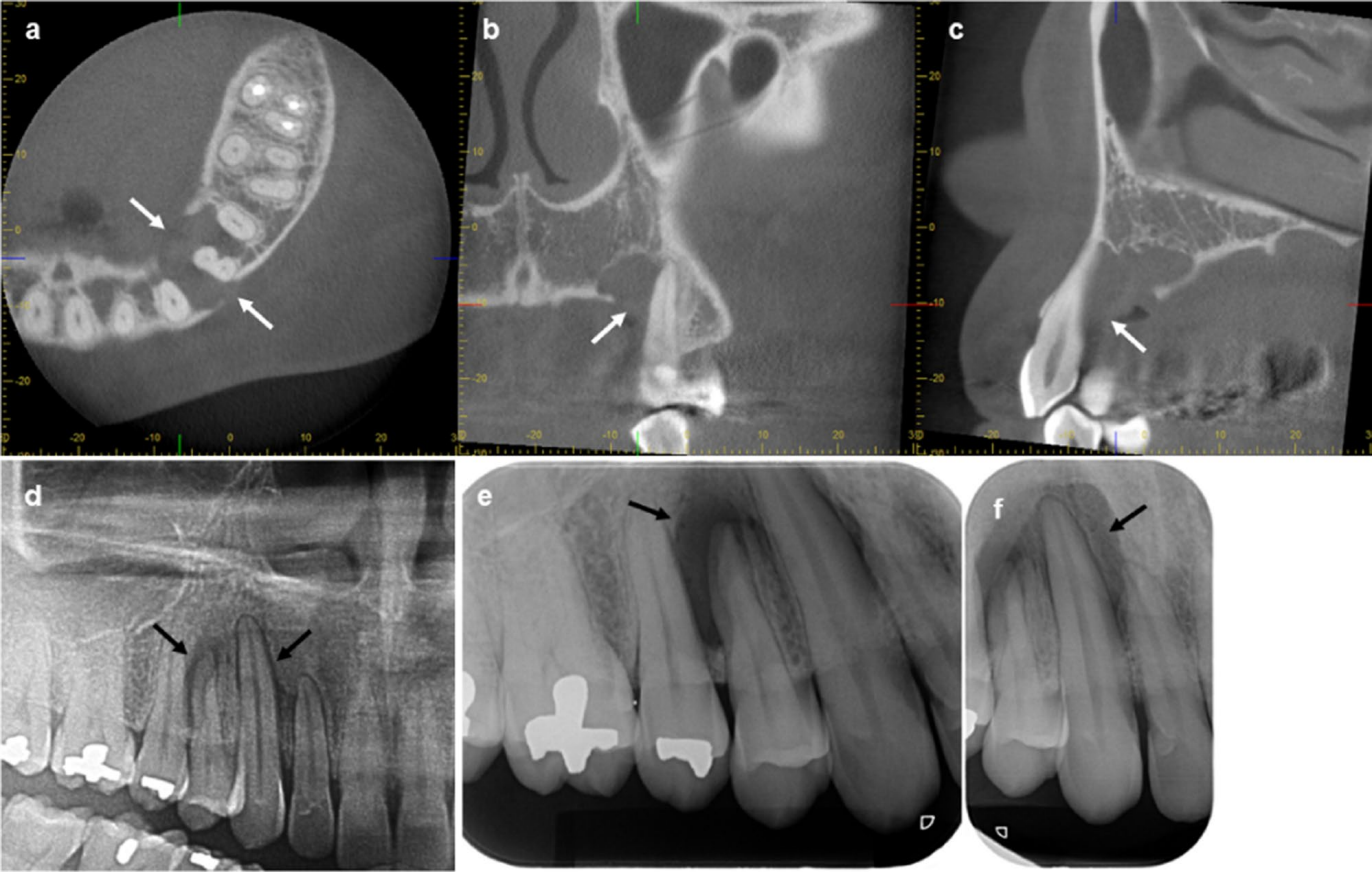
Radiographs of case 1 **(a-c)** and case 2 **(d-f)**. Cone beam computed tomography images in axial view **(a)**, coronal view **(b)** and sagittal view **(c)** show a maxillary lesion with resorption of the bone around teeth without tooth displacement or resorption. Perforation of buccal and palatal cortices (white arrows) is demonstrated without bone expansion. A cropped panoramic image **(d)** and periapical images **(e and f)** show a periapical radiolucent lesion extending from distal surface of the upper right lateral incisor to the mesial surface of the upper right second premolar (black arrows) mimicking periapical inflammatory lesion

**Fig. 2:**
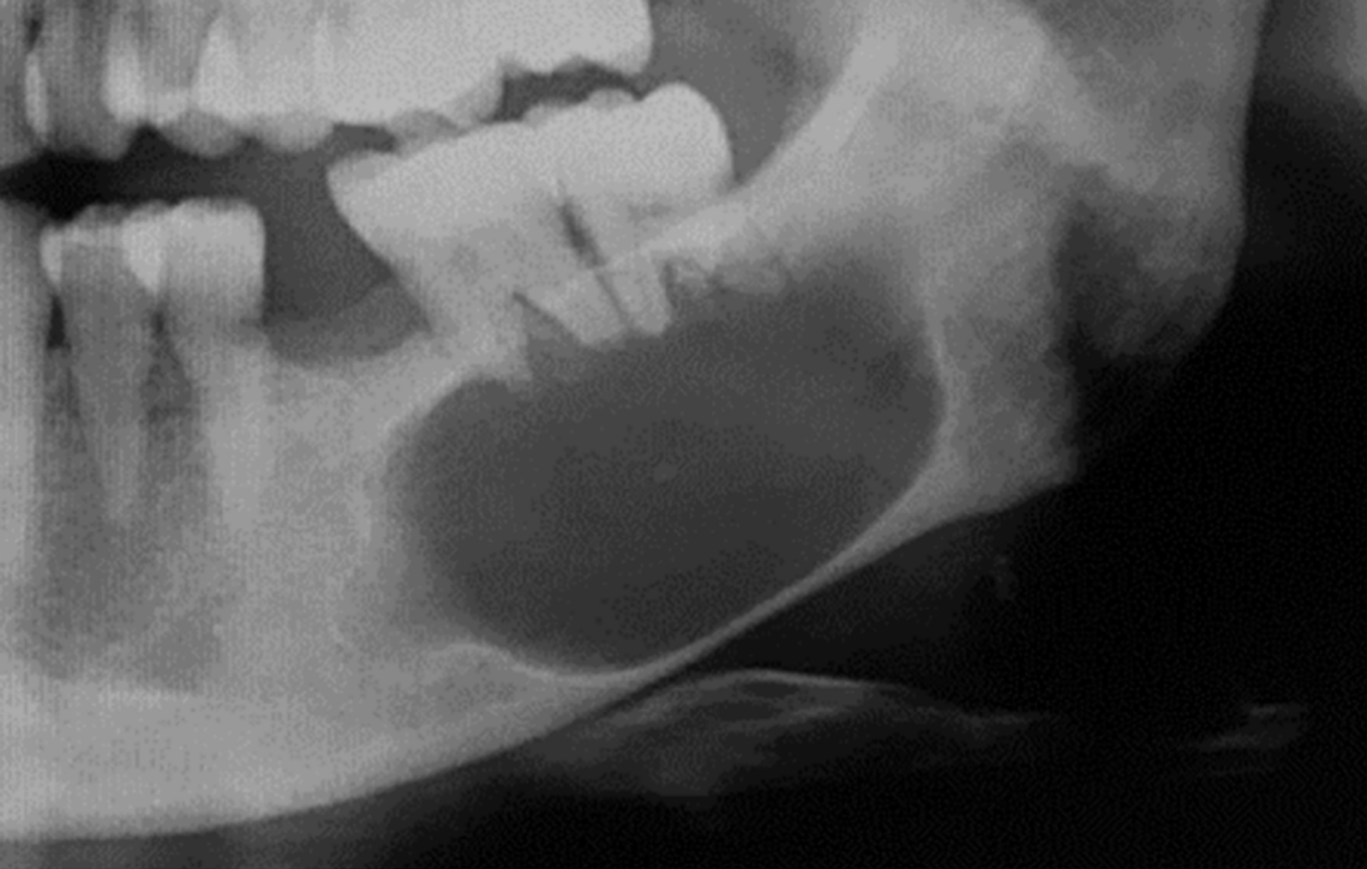
A cropped panoramic image of case 3 shows a unilocular radiolucent lesion extending from the edentulous area of the first molar to the third molar region with root resorption of the second and the third molar

**Fig. 3:**
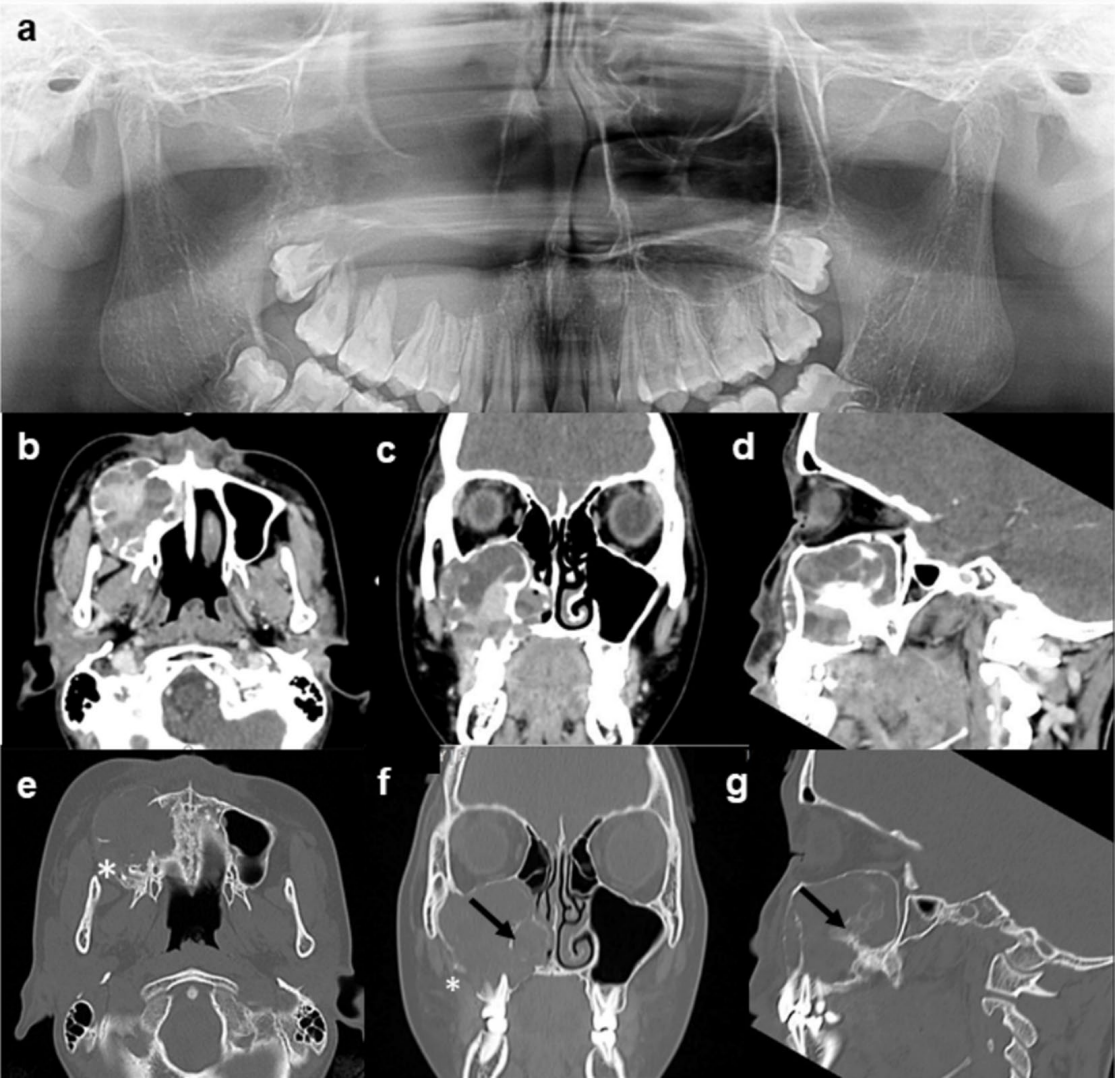
Radiographs of case 5. A cropped panoramic image **(a)**, multidetector computed tomography (MDCT) images with soft tissue window **(b-d)** and MDCT images with bone window **(e-g)** show an expansile mass with heterogeneous enhancement at the right maxilla occupying the right maxillary sinus, right middle and inferior turbinates with expansion of the maxillary sinus walls and area of cortex perforation (*). The lesion shows scalloped border with internal septum (arrows)

**Fig. 4:**
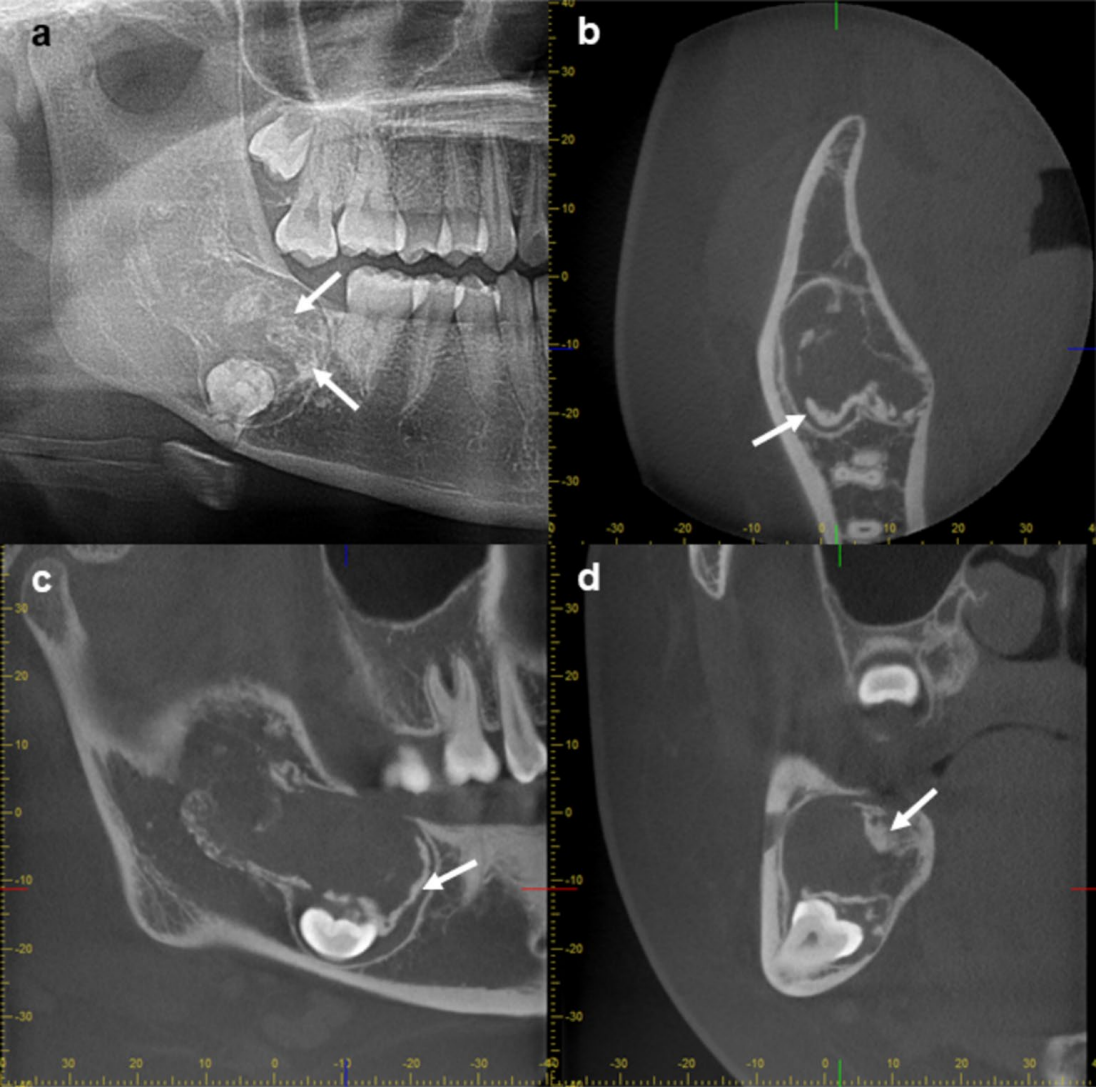
Radiographs of case 6. A preoperative cropped panoramic image **(a)** and cone beam computed tomography images **(b-d)** after incisional biopsy show a low attenuation lesion with internal calcification (arrows) associated with an impacted molar tooth

Radiographic techniques and findings are summarized in Table 2. Four cases showed unilocular radiolucent lesions without associated impacted tooth. One half of the cases showed a smooth border. Cases 1 and 2 (Fig. 1) showed unilocular radiolucent lesions at the anterior maxilla. Cases 3 (Figs. 2) and 4 showed unilocular radiolucent lesions with smooth border at the posterior mandible. The lesion of case 3 extended from the left edentulous area of the lower first molar to the third molar region with root resorption of the lower second and the third molar. Case 4 extended from the lower left second premolar to the mesial root of the lower left second molar. The retained root of the lower left first molar was found associated with the lesion. In case 5 (Fig. 3), an expansile radiolucent lesion was noted extending from the right anterior maxilla to the right posterior region involving the tuberosity. On panoramic image, the lesion was superimposed over the right maxillary sinus. Multidetector computed tomography (MDCT) with contrast enhancement was performed in this case. MDCT images revealed a large lesion with scalloped border and internal septum with heterogeneous enhancement at the right maxilla occupying the right maxillary sinus, right middle and inferior turbinates with expansion of the maxillary sinus walls and area of cortex perforation. The lesion of case 6 showed a scalloped border at the posterior mandible (Fig. 4). The lesion attached to the cemento-enamel junction of an impacted permanent second molar and displaced this tooth inferiorly. Interestingly, this case showed prominent radiopaque masses.

**Table 2:** Radiographic features of 6 cases of central odontogenic fibroma

Case	Radiographic technique	Border	Shape	Density	AIT	Tooth Disp	Root Res	Bone Exp	Bone Perf
1	CBCT	Scallop	Uni	RL	No	No	No	No	Yes
2	Pan, periapical	Smooth	Uni	RL	No	No	No	N/A	N/A
3	Pan	Smooth	Uni	RL	No	No	Yes 2nd, 3rd M	N/A	N/A
4	Pan, Periapical	Smooth	Uni	RL	No	No	No	N/A	N/A
5	Pan, MDCT	Scallop	Multi	RL	No	No	No	Yes	Yes
6	Pan, CBCT	Scallop	Uni	Mixed RL/RP	Yes 2nd M	Yes 2nd M	No	Yes	No

CBCT, Cone beam computed tomography; MDCT, multidetector computed tomography; Pan, panoramic image; Uni, unilocular; Multi, multilocular; RL, radiolucent; RP, radiopaque; AIT, associated impacted tooth; M, molar; Tooth Disp, tooth displacement; Root Res, root resorption; Bone Exp, bone expansion; N/A, no available data; Bone perf, bone perforation

**Fig. 5:**
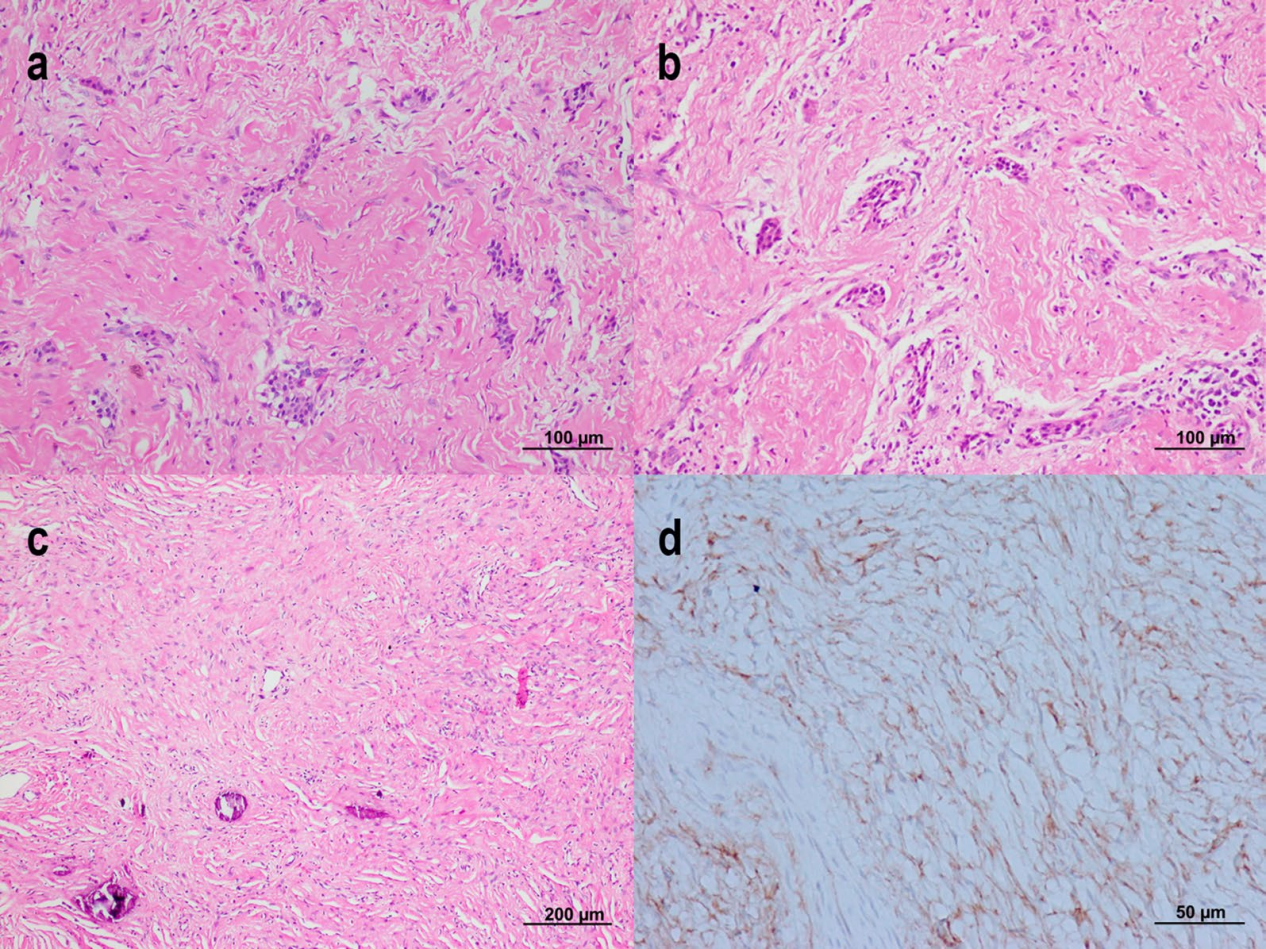
Histopathologic features of case 1 **(a)**, case 2 **(b)** and case 3 (**c** and **d**). **(a and b)** Several odontogenic nests and cords are scattered in a collagenous fibrous connective tissue. **(c)** This tumor shows scattered fibroblasts and foci of calcifications without odontogenic epithelium. **(d)** Immunohistochemical staining shows that CD 99 is expressed in many tumor cells

**Fig. 6:**
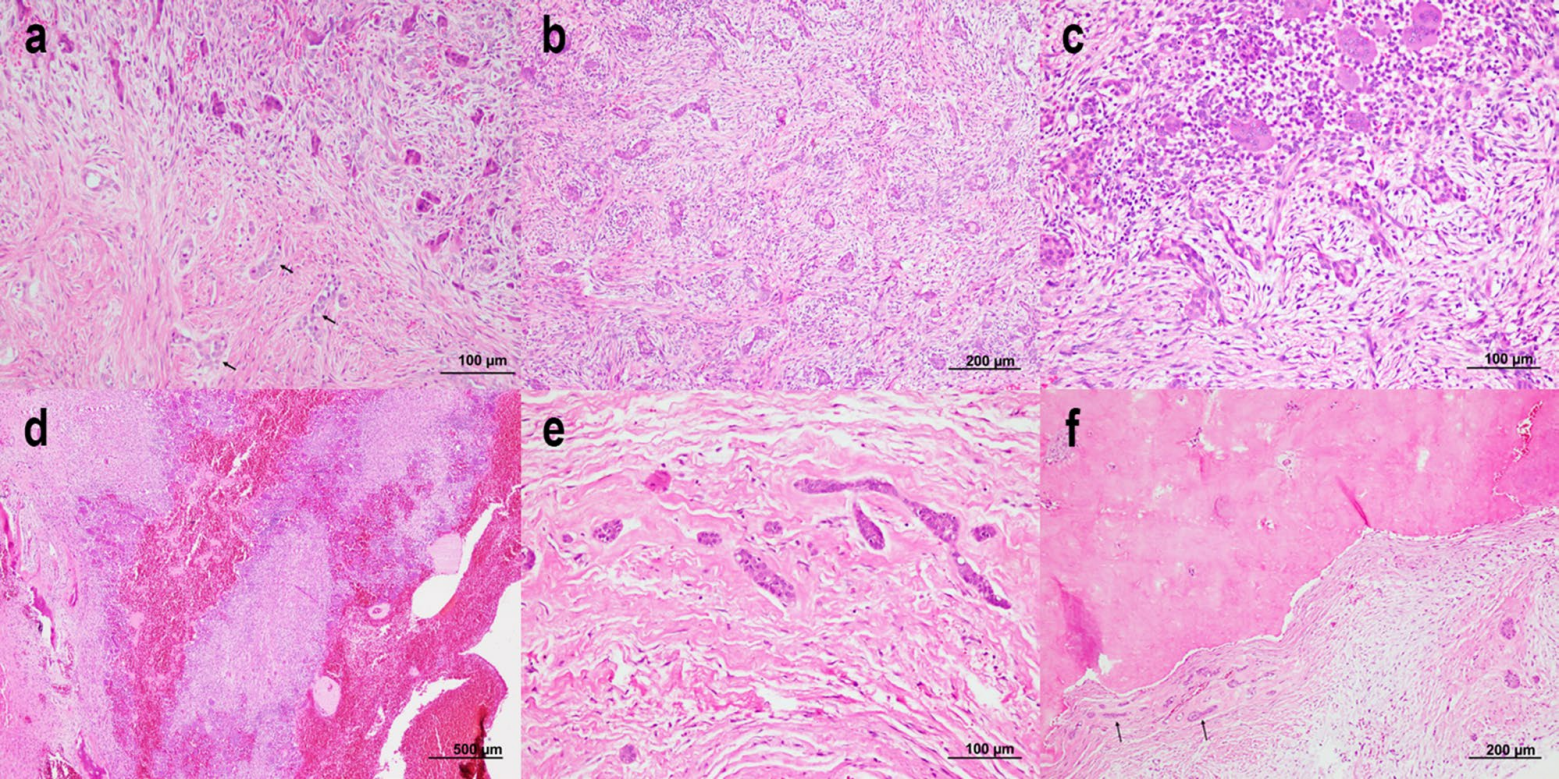
Histopathologic features of case 4 **(a)**, case 5 **(b-d)** and case 6(**e** and **f**). **(a)** The hybrid tumor consists of cords and nests of odontogenic epithelium (arrows) in a fibrous background. The central giant cell granuloma component is seen in the upper part of this figure. **(b)** This area of the triphasic tumor shows the central odontogenic fibroma component. **(c)** This area of the same tumor shows a group of odontogenic rests adjacent to a group of multinucleated giant cells. **(d)** The other area of the same tumor shows prominent blood-filled spaces of the aneurysmal bone cyst component associated with zones of central giant cell granuloma. **(e)** This area of the ossifying subtype shows a collection of odontogenic epithelium in a fibrous stroma. **(f)** The other area of the ossifying subtype contains a large sclerotic mass of cemento-osseous tissue. Note that groups of odontogenic nests and strands (arrows) are observed next to this sclerotic mass

Histopathologic pattern of each case, along with the clinical data, are displayed in Table 1. Cases 1 and 2 shared essential histopathologic features consistent with the epithelium-rich type. They were characterized by a number of nests, small islands and cords of inactive-looking odontogenic epithelium dispersed throughout the moderately cellular fibrous connective tissue (Fig. 5a, b). The stroma was fibromyxomatous in some areas. Scattered chronic inflammatory cells were noticed. A few small calcified masses, resembling woven bone, were detected in case 2. The tumor of case 3 was composed of many spindle-shaped tumor cells. Tumor cells arranged themselves in an interlacing fashion and storiform pattern. Areas of less cellularity in a collagenous background were also observed. A small number of calcifications were focally detected (Fig. 5c). Because the tissue was not decalcified, it became difficult to identify the type of calcified materials in this case. No odontogenic epithelium, foam cells and multinucleated giant cells were discovered. Cellular atypia is not observed. Mitotic activity was hardly detected. Immunohistochemistry was carried out to rule out other benign spindle-shaped tumors. Tumor cells were positive to vimentin and CD99 (Fig. 5d), but negative to S-100, SMA, HHF-35, desmin, CD31, CD34, STAT-6 and pan-cytokeratin (AE1/AE3). Accordingly, the diagnosis of COF was given. The histopathologic features of this case were consistent with the epithelium-poor type. Cases 4 and 5 were associated with CGCG. Case 4 was diagnosed as the hybrid COF with CGCG (Fig. 6a). In addition to the hybrid nature, case 5 (Fig. 6b-d) also contained several small to large blood-filled spaces. Case 5 was considered as the triphasic tumor as this case contained three components within a single tumor. For both cases, areas of COF were found separated from the CGCG component. However, the two components were intermixed in a few areas. The COF component demonstrated numerous nests and strands of odontogenic epithelium in a moderately cellular fibrous connective tissue. The stroma frequently demonstrated a fasciculate or storiform pattern. Zones of CGCG exhibited multiple multinucleated giant cells in a cellular fibrous stroma. Trabeculae of reactive bone and osteoid are often noted particularly in the CGCG component. In case 5, the CGCG component was often associated with blood-filled spaces (Fig. 6d). Because these blood-filled spaces were not lined by endothelial cells, the histopathologic features of these areas were consistent with an aneurysmal bone cyst (ABC). In case 5, the CGCG and ABC components were predominant. The diagnosis of the hybrid COF with CGCG and with secondary ABC was rendered for case 5. The last case was regarded as the ossifying subtype. It consisted of two components: the COF and the calcifying elements. The COF component showed a fibromyxomatous to fibrous tissue embedded with a small number of odontogenic nests or cords (Fig. 6e). These odontogenic epithelial nests tended to cluster together. Foci of dystrophic calcifications and irregular-shaped deposits of basophilic calcified materials were occasionally found in the fibrous stroma. Additionally, several small to large masses of cemento-osseous tissue were detected in close relationship to clusters of odontogenic epithelium (Fig. 6f).

With respect to treatments, all tumors, except for case 5, were totally removed by enucleation with or without additional procedures (Table 1). For each case, the involved tooth or teeth were removed together with the tumor except for case 2. In case 2, no teeth were removed during the tumor enucleation. At the beginning, endodontic treatment was planned for the upper right first premolar. However, this tooth was extracted 15 months after the initial operation due to the dehiscence of the palatal gingiva and deep periodontal pocket with tooth mobility. In case 5, the hybrid COF with CGCG and with secondary ABC, partial block resection, extending from the right second premolar to the third molar, was applied. The lesion in the maxillary sinus was enucleated and the surrounding bone was curettage. However, the ABC component was still identified at some margins according to the histopathologic report. All patients showed no sign of recurrence during the follow-up period ranging from 4 to 33 months.

## Discussion

In this study, six cases of COF were identified from 817 odontogenic tumors and 13,736 specimens during the 16-year period. Thus, COF accounted for only 0.73% of odontogenic tumors and 0.04% among oral and maxillofacial specimens. These relatively low frequencies are in line with previous studies showing that COF is a rare odontogenic tumor. The relative frequency of COF among odontogenic tumors was 0.3 to 2.8% calculated from studies containing a minimum of 1,000 odontogenic tumors [1–4]. Based on three studies consisting of more than 50,000 oral specimens, only 0.02 to 0.03% were diagnosed as COF [1, 3, 4]. Therefore, COF is considered an unfamiliar tumor for oral and maxillofacial pathologists and also other oral healthcare personnel.

The predilection sites for maxillary and mandibular lesions of our series are consistent with prior studies [5, 18]. In our series, all lesions in the mandible were found in the molar area whereas two cases in the maxilla occurred anterior to the first molar. One of the maxillary lesions in this study was a large expansile lesion extending from the anterior part of the maxilla to the tuberosity and occupying the entire maxillary sinus, reflecting its aggressive behavior. Interestingly, this case showed a unique histopathologic feature. It was the hybrid COF with CGCG in conjunction with secondary ABC. This kind of histopathologic appearance has never been documented. The intra-oral manifestation of one maxillary lesion (case 2) demonstrated a palatal depression. Several authors have noticed palatal depressions over maxillary COF [7, 8]. According to the latest systematic review, mucosal depression and mucosal perforation or fistula were found in 8.9 and 3.7% of palatal tumors, respectively [5]. In an international multicentric study of 62 COF, palatal depression was detected in 9 cases (27.3%) from 33 maxillary tumors [9]. Taken altogether, we believe that these oral manifestations are quite distinct from other tumors or cysts occurring at the maxilla and may be a useful sign for making a clinical diagnosis of COF at this area.

The radiographic analysis of 120 COF from a systematic review has shown that unilocular radiolucent lesions (54%) were the most common radiographic features, followed by multilocular radiolucent lesions (24%) [5]. In accordance to this systematic review, our study found that four out of six cases showed a unilocular radiolucent appearance whereas one case showed a multilocular radiolucent pattern. Several investigators have found that most COF are totally radiolucent, although a mixed radiolucent-radiopaque appearance has been reported in about 11% of COF [5]. Consistent with a systematic review, our series found that only one from six cases (16.7%) exhibited prominent radiopaque masses. With respect to association with impacted tooth, 4.7% of COF showed this kind of association [18]. In our study, one COF was associated with an impacted second molar. Previous works have shown that tooth displacement was observed between 40 and 55% while root resorption was found between 24 and 46% [5, 9]. However, another systematic review reported a relatively low frequency of tooth displacement with the percentage of 4.1% from 173 examined COF [18]. Therefore, it remains to be determined whether tooth displacement is common in COF. In this study, tooth displacement (case 6) and root resorption (case 3) were observed in only one case each.

With respect to radiographic differential diagnosis, making a diagnosis of COF is difficult due to its rarity and its variability as previously discussed. If the lesion presents as a periradicular radiolucent lesion similar to cases 1 and 2, differential diagnosis includes lateral periodontal cyst, odontogenic keratocyst and squamous odontogenic tumor. Additionally, if the involved teeth are non-vital, lesions related to periapical inflammation such as periapical granuloma or radicular cyst might be included in the differential diagnosis. In cases 3 and 4, the lesions were located at the apical region of premolar and molar teeth of the mandible. Therefore, ameloblastoma and odontogenic keratocyst should be in the list of differential diagnosis. Case 5 was an aggressive lesion at the posterior maxilla extending into the maxillary sinus. Although the radiographic features showed considerable size and severe bone destruction, the margin of the lesion was well-demarcated without penetration into adjacent soft tissue. Hence, the differential diagnosis of benign aggressive lesions or tumors, i.e., ABC, CGCG and ameloblastoma was made. Notably, COF with huge size and aggressive behavior, like case 5 in this study, is rare. Recently, an aggressive COF at the posterior maxilla in a 53-year-old man has been reported [19]. The provisional diagnosis for case 6, a 14-year-old female presented with internal large calcifications, were ameloblastic fibro-odontoma or developing odontoma, calcifying odontogenic cyst, adenomatoid odontogenic tumor and calcifying epithelial odontogenic tumor. Internal radiopacities in COF have been reported in about 9 to 18% of COFs [5, 9, 18]. Calcifications in radiographs of COF were presented as small radiopacities or large radiopaque masses [20–23]. In some COF, calcifications are very prominent and may be difficult to separate from fibro-osseous lesions [20, 24].

According to the latest systematic review, the epithelium-rich and the epithelium-poor types were found in almost equal proportion with percentages of 34.1 and 28.9%, respectively [5]. It is well-accepted that COF absence from odontogenic epithelium is also regarded as COF [10–12]. However, COF without odontogenic epithelium was demonstrated by only a few groups in the English-language literature [10, 11]. They diagnosed their cases as COF, the simple type without immunohistochemical analysis. Similar to a related case [11], case 3 in this study demonstrated many spindle-shaped cells arranged in a storiform pattern or interlacing bundles. Foci of calcifications were evidenced. Before establishing a diagnosis of COF, several tumors containing uniform spindle-shaped cells such as desmoplastic fibroma, leiomyoma, solitary fibrous tumor and tumors of neural origin, must be excluded. Therefore, we performed immunohistochemical staining to rule out these lesions. Tumor cells in case 3 of our study were positive to vimentin and CD99 but they were negative to S-100, desmin, HHF-35, SMA, CD31, CD34, STAT-6 and pan-cytokeratin (AE1/AE3). The negative results to S-100 and desmin as well as HHF-35 excluded tumors of neural and muscle origins. The diagnosis of solitary fibrous tumor and tumors of endothelial origin were excluded by the negativity of tumor cells for STAT-6, CD34 and CD31. The reason to use an antibody against pan-cytokeratin was to search for odontogenic epithelium that might be missed during the examination of hematoxylin and eosin slides. The negativity of tumor cells to SMA disfavored the diagnosis of desmoplastic fibroma [25]. The positive reactivity of tumor cells to CD99 in our study is in line with a previous study. One related work demonstrated the positivity of some fibroblast-like cells for CD99 in COF [26]. They explained that the expression of CD99 in COF may be due to the neuroectodermal origin of this tumor. The separation of the epithelium-poor type of COF from desmoplastic fibroma is of clinical importance as the latter is a locally aggressive benign tumor with high recurrence. It has been proposed that the presence of irregular small calcifications favors the diagnosis of COF whereas broader and longer bands of collagen fibers advocate desmoplastic fibroma [27]. In our opinion, in addition to the histopathologic examination, immunohistochemical analysis should be performed to distinguish these two fibroblastic tumors.

Case 4 in our study was a hybrid COF with CGCG. This case was previously reported by our colleague in 2019 at six months follow-up with uneventful results [28]. The patient was lost to follow-up in 2021. Thus, the patient was monitored for 19 months without evidence of recurrence. So far, 50 cases of this variant have been reported in the articles published in English language [29]. Except for the occurrence in male patient, the clinical, radiographic and histopathologic features of the hybrid COF with CGCG of our study were similar to previous hybrid cases. Three hypotheses have been proposed to be the pathogenesis of the hybrid COF with CGCG. First, this variant is a true collision of COF and CGCG. The second theory postulated that the COF is the primary lesion, in which trauma or other injuries induce a giant cell reaction. In contrast to the second theory, the third theory proposes that the CGCG is the primary lesion that triggers the COF component [30, 31]. Despite the controversy over the pathogenesis of this variant, it is well known that the hybrid COF with CGCG is more aggressive and has a higher recurrent rate than the conventional COF [32]. The treatments of this hybrid may be similar to CGCG. These include thorough curettage or conservative surgical excision. A close follow-up is highly advised [32]. Additionally, it has to be aware that the histopathologic features of the CGCG component are indistinguishable from the brown tumor of hyperparathyroidism. Thus, serological investigations may be considered in patients diagnosed as hybrid COF with CGCG.

The tumor of case 5 in this study was composed of COF, CGCG and ABC components within a single tumor. The triphasic characteristic of COF has never been reported. ABC is an intra-osseous lesion consisting of several blood-filled spaces of variable size separated by fibrous stroma coupled with trabeculae of woven bone and multinucleated giant cells. Based on the clinicopathologic features, ABC can be categorized into primary and secondary types. Primary ABC has been proposed to be a true neoplasm as it contains rearrangements of *CDH*11 and *USP*6 genes. However, secondary ABC does not show oncogenic alterations and therefore is considered to be the actual pseudocyst as a result of a reactive process [33]. Only 1.8% of ABC are discovered in the jaw [34]. In this location, secondary ABC has been reported between 15 and 76%. Among the associated lesions with secondary ABC, fibro-osseous lesions were the most common lesions, followed by CGCG [35]. The pathogenesis of case 5, the triphasic tumor, is a matter of speculation. We postulate that the hybrid COF with CGCG is the initial lesion, followed by the development of ABC. This is supported by the fact that primary CGCG either with or without ABC is more commonly found in the mandible, while COF tends to occur in the anterior maxilla. In our study, the tumor of case 5 extended from the right anterior maxilla to the right posterior region involving the maxillary sinus. This triphasic tumor poses a challenging issue with respect to the diagnosis, treatment and prognosis. The posterior maxilla is considered to be a rare location for COF, CGCG and ABC as individual lesion or as the hybrid lesions. Therefore, making a diagnosis is challenging for both clinical and histopathologic aspects. The histopathologic examinations of the first and the second biopsies of case 5 only showed the hybrid COF with CGCG. The ABC component was finally revealed from the excisional biopsy. The definite diagnosis of this case was given after three times biopsies, suggesting the difficulty in the microscopic diagnosis of this rare case. Due to the complex anatomy in the maxilla, completely removing this lesion is also difficult, leading to a high tendency to recur. Thus, long-term monitoring of this patient is necessary.

Case 6 was regarded as the ossifying subtype. The ossifying variant has been introduced as a distinct subtype in the 2022 WHO classification [4, 6]. Based on related studies, only nine cases of this variant have been reported [7, 9, 24]. Thus, our case is considered to be the tenth case of the ossifying variant of COF. In three of nine cases, the ossifying variant has been described as COF with ossifying fibroma-like tissue [7, 24], whereas the characteristics of calcifications in the remaining six cases were not mentioned. In contrast to previous cases, the ossifying variant of the present study contained materials resembling dentinoid, which is not expected in this variant of COF. Additionally, its pericoronal location with extensive calcification also represents a rare radiographic characteristic for COF. Interestingly, two prior cases of COF with mixed radiolucent-radiopaque radiographs contained prominent cemento-osseous tissue similar to our case [22, 36]. Although these cases were not reported as the ossifying variant by the authors at that time, we wonder whether these cases may be the ossifying subtype of COF. Therefore, the number of the ossifying subtype may be undercounted. Importantly, the criteria to designate COF as the ossifying subtype are required. Furthermore, we encourage reporting more cases of the ossifying variant along with details regarding the characteristics of calcifications.

In terms of treatment for COF, many studies suggest a conservative surgery including enucleation and curettage [5, 7, 9, 17, 18]. Selective extraction of involved teeth with prominent root resorption was considered [7]. Nevertheless, there was no statistically significant correlation between the extraction of involved teeth and recurrence [17]. It has been shown that higher recurrence rate is associated with maxilla location, cortical bone perforation and multilocular radiolucency [18]. Recurrent rate has been reported between 2.9 and 10% [9, 17, 18]. The minimum follow-up period of 60 months was recommended [5]. Malignant transformation has never been documented. Nevertheless, it has to be aware that the histopathologic features of COF may be difficult to separate from sclerosing odontogenic carcinoma (SDC). SDC is a rare malignant odontogenic carcinoma. It is composed of strands and nests of bland odontogenic epithelium in a dense sclerotic stroma. Unlike COF, SDC shows infiltrative growth and invasion of skeletal muscle and nerves [6, 14].

## Conclusions

Six cases of COF with diverse radiographic and histopathologic appearances were reported. The triphasic tumor consisting of the COF, CGCG and ABC, is reported here for the first time, while the ossifying subtype is considered the tenth case reported in the English-language literature. Oral and maxillofacial pathologists and other healthcare personnel must be aware of this rare odontogenic tumor and its variants to achieve the definite diagnosis. More cases of COF along with radiographic and histopathologic details of each variant need to be reported to better understand this tumor, leading to the adequate treatment for the patients.

## Data Availability

The data used to support the findings of the study are included in the article.

## Abbreviations

ABC: Aneurysmal bone cyst
CD: Cluster of differentiation
CGCG: Central giant cell granuloma
COF: Central odontogenic fibroma
EDTA: Ethylenediaminetetraacetic acid
MDCT: Multidetector computed tomography
SDC: Sclerosing odontogenic carcinoma
SMA: Smooth muscle actin
S-100: Soluble in saturated (100%) ammonium sulfate solution
STAT-6: Signal Transducer and Activator of Transcription 6
WHO: World Health Organization
